# REEXAMINING RACE AND ETHNICITY FROM A STRUCTURAL RACISM AND SOCIAL DETERMINANTS OF HEALTH LENS IN COHORT STUDIES

**DOI:** 10.1093/geroni/igac059.2919

**Published:** 2022-12-20

**Authors:** Shawna Follis

**Affiliations:** Stanford University, San Francisco, California, United States

## Abstract

To advance research on the role of race, ethnicity, and structural racism in health disparities, recent calls-to-action have described methods to correct race and ethnicity categorization and to measure structural and social determinants of health (SSDOH). Following the rapid advance in the understanding of race and SSDOH, long running cohort studies typically lack access to this novel data, which has limited SSDOH evidence to cross-sectional and ecological associations. We implemented methods to rectify this data gap in prospective cohort studies, using The Women’s Health Initiative (WHI) cohort of women age 50–79 years at baseline as a case study. We evaluated the quality, precision, and representativeness of race, ethnicity, and SSDOH data compared with the target US population and operationalized methods to quantify structural determinants in cohort studies. The use of a revised race and ethnicity categorization aligned with theory-based recommendations and improved measurement precision, including decreasing missing data and participants reporting “other” race. The revised categorization was harmonized to the US Census permitting description of the generalizability of WHI race and ethnicity groups in relation to the target population of US women. We found that WHI women reflected broader trends to the target population in relation to structural racism between race and ethnicity groups, while mean rates of SSDOH were systematically more advantageous in WHI. These results underscore the need to implement theoretically justified race and ethnicity measurement to accurately investigate the intersectional role of SSDOH underlying racial and ethnic health disparities among older age women.

